# Accuracy of online survey assessment of mental disorders and suicidal thoughts and behaviors in Spanish university students. Results of the WHO World Mental Health- International College Student initiative

**DOI:** 10.1371/journal.pone.0221529

**Published:** 2019-09-05

**Authors:** Laura Ballester, Itxaso Alayo, Gemma Vilagut, José Almenara, Ana Isabel Cebrià, Enrique Echeburúa, Andrea Gabilondo, Margalida Gili, Carolina Lagares, José Antonio Piqueras, Miquel Roca, Victoria Soto-Sanz, Maria Jesús Blasco, Pere Castellví, Carlos G. Forero, Ronny Bruffaerts, Philippe Mortier, Randy P. Auerbach, Matthew K. Nock, Nancy Sampson, Ronald C. Kessler, Jordi Alonso, on behalf of the UNIVERSAL study group

**Affiliations:** 1 Health Services Research Group, IMIM (Institut Hospital del Mar d´Investigacions Mèdiques), Barcelona, Spain; 2 Girona University (UdG), Girona, Spain; 3 CIBER Epidemiología y Salud Pública (CIBERESP), Madrid, Spain; 4 University of Cádiz (UCA), Cádiz, Spain; 5 Department of Mental Health, Corporació Sanitaria Parc Taulí, Sabadell, Spain; 6 CIBER Salud Mental (CIBERSAM), Madrid, Spain; 7 University of the Basque Country (UPV-EHU), San Sebastián, Spain; 8 BioDonostia Health Research Institute, Osakidetza, San Sebastián, Spain; 9 InstitutUniversitarid’Investigació en Ciències de la Salut (IUNICS-IDISPA), University of Balearic Islands (UIB), Palma de Mallorca, Spain; 10 Miguel Hernandez University of Elche (UMH), Elche, Spain; 11 University of Jaén (UJA), Jaén, Spain; 12 International University of Catalonia (UIC), Barcelona, Spain; 13 UniversitairPsychiatrisch Centrum, KULeuven (UPC-KUL), Center for Public Health Psychiatry, KULeuven, Leuven, Belgium; 14 Department of Psychiatry, Columbia University, New York, New York, United States of America; 15 Department of Psychology, Harvard University, Boston, Massachusetts, United States of America; 16 Department of Health Care Policy, Harvard Medical School, Boston, Massachusetts, United States of America; 17 PompeuFabraUniversity (UPF), Barcelona, Spain; Department of Psychiatry and Neuropsychology, Maastricht University Medical Center, NETHERLANDS

## Abstract

**Objective:**

To assess the accuracy of WMH-ICS online screening scales for evaluating four common mental disorders (Major Depressive Episode[MDE], Mania/Hypomania[M/H], Panic Disorder[PD], Generalized Anxiety Disorder[GAD]) and suicidal thoughts and behaviors[STB] used in the UNIVERSAL project.

**Methods:**

Clinical diagnostic reappraisal was carried out on a subsample of the UNIVERSAL project, a longitudinal online survey of first year Spanish students (18–24 years old), part of the WHO World Mental Health-International College Student (WMH-ICS) initiative. Lifetime and 12-month prevalence of MDE, M/H, PD, GAD and STB were assessed with the Composite International Diagnostic Interview-Screening Scales [CIDI-SC], the Self-Injurious Thoughts and Behaviors Interview [SITBI] and the Columbia-Suicide Severity Rating Scale [C-SSRS]. Trained clinical psychologists, blinded to responses in the initial survey, administered via telephone the Mini-International Neuropsychiatric Interview [MINI]. Measures of diagnostic accuracy and McNemar χ^2^ test were calculated. Sensitivity analyses were conducted to maximize diagnostic capacity.

**Results:**

A total of 287 students were included in the clinical reappraisal study. For 12-month and lifetime mood disorders, sensitivity/specificity were 67%/88.6% and 65%/73.3%, respectively. For 12-month and lifetime anxiety disorders, these were 76.8%/86.5% and 59.6%/71.1%, and for 12-month and lifetime STB, 75.9%/94.8% and 87.2%/86.3%. For 12-month and lifetime mood disorders, anxiety disorders and STB, positive predictive values were in the range of 18.1–55.1% and negative predictive values 90.2–99.0%; likelihood ratios positive were in the range of 2.1–14.6 and likelihood ratios negative 0.1–0.6. All outcomes showed adequate areas under the curve [AUCs] (AUC>0.7), except M/H and PD (AUC = 0.6). Post hoc analyses to select optimal diagnostic thresholds led to improved concordance for all diagnoses (AUCs>0.8).

**Conclusion:**

The WMS-ICS survey showed reasonable concordance with the MINI telephone interviews performed by mental health professionals, when utilizing optimized cut-off scores. The current study provides initial evidence that the WMS-ICS survey might be useful for screening purposes.

## Introduction

According to the World Health Organization, based on a systematic review and meta-analysis carried out (n = 829,673 from 63 countries; age-range = 16–65 years old), the population 12-month prevalence of mental disorders is 17.6% [1]. At the same time, estimates based on data from 28 countries throughout the world (n = 85,052; age = 18 years old or more), indicate a 12-month prevalence of 9.8–19.1% (interquartile range, 25th–75th percentiles across countries) in the general adult population[2]. Many mental disorders (phobias and impulse-control disorders) have an early age of onset (before 15 years old) and others (mood, anxiety and alcohol) have a peak period during college years [3,4]. Mental disorders with early manifestation might become chronic if not effectively treated [5–7].

Thus, research in the young population is clearly needed to develop better epidemiological approaches to diminish the burden of mental disorders[8]. University students make up a significant fraction of the population younger than 25 in developed countries [9]. Epidemiological studies suggest that mental disorders and suicidal thoughts and behaviors are common among university students, and that less than 25% of individuals with a mental disorder sought treatment in the year prior to the survey[10–12].

Screening instruments for the assessment of mental disorders are valuable for providing accurate measurements [13,14] as well as the accessibility to brief and simple tools that can facilitate the investigation of mental disorders[15]. Some studies have demonstrated that self-administered instruments show good psychometric properties in younger and middle-aged adults, such as the General Health Questionnaire (GHQ) vs. interviewer-administered version of the Clinical Interview Schedule-Revised (CIS) (Sensitivity = 72.2, Specificity = 78.0, Positive Predictive Value = 40.0, Negative Predictive Value = 93.4)[16]. Another study that evaluated psychometric properties of a questionnaire for screening people with anxiety/depression self-administered vs. interviewer-administered, self-administered version showed high sensitivities (87.0–92.0) and PPVs (86.0–87.0), but lower specificities (29.0–45.0) and NPVs (38.0–50.0)[17]. Also, self-administered and interviewer-administered versions of the Composite International Diagnostic Interview (CIDI) showed good kappa agreement[18].

Self-administered computerized assessments have great potential for screening mental disorders in different settings [19]. Self-administered computerized assessments of mental disorders have been developed with similar ascertainment of morbidity as when identical questionnaires are administered by an interviewer [19]. Self-administered instruments permit participants to respond more truthfully than in interviewer-administered assessments without social desirability bias [20]. Another significant advantage of self-administered instruments is their brevity and ease of administration, which facilitates assessing mental disorders in epidemiologic studies [21–24].

The UNIVERSAL project, a part of the World Mental Health International College Surveys (WMH-ICS) initiative [25], is a multi-center, cohort study to assess the prevalence and incidence of mental disorders and suicidal thoughts and behaviors, as well as to identify the main risk factors and associated protectors among Spanish university students[26]. The online survey of UNIVERSAL and WMH-ICS include screening scales for the assessment of mental disorders derived from the WHO Composite International Diagnostic Interview (CIDI)[27] and the Composite International Diagnostic Interview Screening Scales (CIDI-SC) [28]. In addition, suicidal thought and behavior items are assessed using items derived from the Self-Injurious Thoughts and Behaviors Interview (SITBI) [29]and the Columbia-Suicidal Severity Rating Scale (C-SSRS)[30]. The concordance of CIDI screening scales (CIDI-SC) with the Structured Clinical Interview from DSM-IV (SCID) was exhaustively evaluated showing good individual-level concordance between the two instruments among active duty Army personnel [13]. But diagnostic accuracy remains untested in samples of college students [25].

The objective of this study is to assess the diagnostic capacity of the WMH-ICS online survey screeners for four common mental disorders (Major Depression Episode [MDE], Mania/Hypomania [M/H], Panic Disorder [PD], and Generalized Anxiety Disorder [GAD]) and for Suicidal Thoughts and Behaviors [STB] among university students.

## Methods

### The UNIVERSAL study

The UNIVERSAL project is part of the World Mental Health International College Student (WMH-ICS) initiative for the study of mental disorders among first-year college students (https://www.hcp.med.harvard.edu/wmh/college_student_survey.php). More detailed description of the WMH-ICS initiative can be found elsewhere[25,31,32]. UNIVERSAL is a multi-center, observational cohort study of all students starting their 1^st^ course in 5 Spanish universities from 5 Spanish autonomous regions (Andalusia, Balearic Islands, Basque Country, Catalonia and Valencia). A total of 2,343 incoming first year students, during the 2014/15 academic year, were recruited for the study and answered the online baseline survey. Inclusion criteria for eligible students at baseline were: (i) age range from 18 to 24 years old; and (ii) first time enrolment at a university degree. The students participating in the study were re-contacted every year, from 2015/16 to 2017/18 courses, for follow-up online assessments.

Students were invited to complete the study registration form through the UNIVERSAL website (https://www.upf.edu/web/estudiouniversal; https://encuesta.estudio-universal.net) and after agreeing with the informed consent, they were asked to provide personal contact information so they could be re-contacted to complete the survey. The data collection platform follows the international recommendations and guidelines for computerized assessment (International Test Commission -ITC-, 2005)[33]. Further information on the UNIVERSAL project has been published elsewhere[32].

### Clinical reappraisal sample

A clinical reappraisal study of a subsample of university students participating in the UNIVERSAL project was carried out. After responding to the online survey, a sub-sample of eligible students was invited to participate in a telephone clinical interview using the Mini International Neuropsychiatric Interview (MINI)[14]. Eligibility for the clinical reappraisal sub-study was determined by whether individuals: (i) provided a contact telephone number available; (ii) completed informed consent to participate in the reappraisal study; and (iii) completed the diagnostic sections of the online screeners (i.e., for the baseline sub-sample the lifetime and 12-month prevalence was evaluated and for the sub-sample recruited from the 1^st^ and 2^nd^ follow-up the 12-month prevalence was assessed).

Eligible students were selected for the clinical reappraisal sub-study at different time periods of the baseline and follow-up assessments. Consecutive sampling of cases was applied for students reappraised at baseline (starting in May 2015, academic course 2014/2015) and 1^st^ year follow-up assessment (academic course 2015/16, starting in March). For the second year of follow-up (academic course 2016/17, starting selection in November 2016) the method of recruitment of the subjects interviewed was modified to assure sufficient number of individuals with a disorder. To preserve the possibility of restoring the original distribution of the online survey sample, a probabilistic selection was carried out, with over-sampling of students who screened positive in the corresponding online screeners. Specifically, we selected 100% of those who screened positive in any of the following GAD, PD, M/H, suicide plan, and suicide attempt; 20% of individuals with MDE or suicidal ideation (but none of the above); and, 10% of the rest of the sample were selected. S9 Table shows prevalence estimates in each reappraisal sample, selected in each follow-up to carry out the reassessment.

Eligible students were systematically invited by telephone and asked for consent to participate in the re-appraisal interview within 4 weeks of completing the online survey whether it was at baseline, 1^st^ year follow-up assessment or 2^nd^ year of follow-up. They were blind to the results of the online survey responses. At least 5 phone call attempts were made on different days of the week and hours of the day. If a participant could not be contacted, he/she was considered missing for the clinical reappraisal.

### Online screening measures

The online survey used in this project gathers self-reported data about mental health and a wide range of possible risk and protective (i.e., sociodemographic, general health, mental wellbeing, mental disorders, STB, use of services, stressful life events). Overall, the survey was composed of 291 items, but includes logical skips in the symptomatology section according to the students’ response to shorten the length of the survey. The mean time for completion of the survey was 39 min (SD = 8 min; Pc25 = 33 min—Pc75 = 45 min).

The online survey included short self-report screening scales for lifetime and 12-month prevalence of four common disorders (MDE, M/H, GAD, and PD). This subset of four disorders of the WMH-ICS surveys is associated with the highest levels of role impairment among college students in the WMH surveys[25]. The items were based on the Composite International Diagnostic Interview Screening Scales (CIDI-SC)[13,27,28], an integrated series of multi-lingual diagnostic screening scales chosen for their good psychometric properties[13] and concordance with clinical diagnoses[25]. The online survey also included assessment of STB based on the Columbia-Suicidal Severity Rating Scale (C-SSRS)[30] and the Self-Injurious Thoughts and Behaviors Interview (SITBI)[29] instrument that has been translated to Spanish as the “*Escala de Pensamientos y ConductasAutolesivas”* (EPCA) [34], showing good clinical diagnosis concordance in Spanish adult psychiatric patients (mean age = 43.3 years) [34].

Screening scales diagnostic algorithms from the ARMY STARRS survey were adapted for their use in the WMH-ICS self-administered questionnaire [12]. More information about characteristics of the survey was published by Blasco et al. (2016) [32].

### Clinical reappraisal interview

The Spanish MINI 5.0.0 [35] and 6.0[36] for mental disorders and suicidal thoughts and behaviors were administered in the re-appraisal interview. The MINI is a structured interview that assesses DSM-IV-TR axis I mental disorders[37], and one of its major advantages is the short administration time [mean (SE) 18.7(11.6) minutes] [38]. For most mental disorders, the MINI shows values higher than 0.70 for sensitivity (SN) and 0.85 for specificity (SP) in relation to the Structured Clinical Interview for DSM-III-R Patients (SCID-P) [38]. In relation to psychiatrist’s diagnostic judgement, the Spanish MINI shows values higher than 0.90 for SN and 0.60 for SP for most mental disorders[35].

For consistency with the online survey recall periods, we added a 12-months assessment period together with lifetime assessment in corresponding sections of the MINI structured interview for all disorders evaluated. Since telephone vs. in-person modes seem not to influence findings [39–42], interviews were performed via telephone. Interviewers were blind to the online survey responses, and no personal information (other than telephone) was provided to them.

Re-appraisal interviews were performed by seven clinical psychologists with a range of 1 to 15 years of clinical experience. Two senior clinical psychologists developed the protocol to perform the MINI telephone interview in a standardized way. Also, a registry was created to introduce dates of five phone call attempts with students and the reason of refused/fail contact. The experts supervised *in situ* the first five to ten interviews carried out by the each interviewer to ensure standardized procedures were satisfactorily followed.

### Analysis

As noted earlier, diagnostic algorithms used in the present study are taken from the ARMY STARRS survey. We compared lifetime and 12-month prevalence estimates among the overall sample and the reappraised sub-sample according to the online screening index tests using chi-squared test. The McNemar χ^2^ test was also calculated for evaluating the prevalence differences between index test diagnosis and reference standard.

Agreement was assessed by comparing each online screening index test diagnosis with the reference standard (MINI). Estimates of disaggregated measures were performed: Sensitivity SN (% of reference standard cases detected by the index test), Specificity SP (% of reference standard non-cases correctly classified as non-cases by the index test), Positive Predictive Value PPV (% of index test cases confirmed by the reference standard), Negative Predictive Value NPV (% of index test non-cases confirmed as non-cases by the reference standard) and likelihood ratio positive LR+ (proportion of reference standard cases testing positive according to the index test divided by the proportion of non-cases testing positive in the index test) and likelihood ratio negative LR- (proportion of reference standard cases testing negative divided by the proportion of non-cases testing negative in the index test). Likelihood ratio is a constant value and can be used to arrive at a posttest probability, which facilitates appraising how a particular test result predicts the risk of disease [43,44]. Receiver Operating Characteristics (ROC) analyses were performed for diagnostic capacity of the instruments, including area under the curve (AUC), considering the MINI diagnoses as the reference standard. Labels of agreement were assigned to the different ranges of AUC according to Landis and Koch as slight (0.50–0.59), fair (0.6–0.69), moderate (0.7–0.79), substantial (0.8–0.89) and almost perfect (≥0.9) [13,45]. The AUC can be used between a dichotomous predictor and a dichotomous outcome, where AUC equals (SN+SP)/2[40].

Inverse probability weighting was applied to adjust for the sampling method applied in the reappraisal selection carried out during the 3^rd^ year follow up (2016/17). Weights were obtained as the inverse of the probability of selection within each stratum in 3^rd^ year follow up and normalized to the total sample size of the clinical reappraisal study. Post-stratification weights were calculated and applied in order to correct for imbalances of gender, academic field and nationality characteristics between the clinical reappraisal sample and their respective UNIVERSAL sample, as their reference population. Analysis were performed using SAS v9.4 [46] and SPSS v23.0 [47].

### Sensitivity analyses to improve diagnostic accuracy

Sensitivity analyses were performed for specific disorders of MDE, M/H, PD and GAD to evaluate potential improvements of diagnostic capacity by modifying cut-off points of diagnostic algorithms. We present results to improve diagnostic accuracy according to two different criteria, given that the most useful cut-off points in screening scales may differ depending on the objectives and purpose of the study[13]. For instance, an epidemiological study could prioritize the accurate estimation of the gold standard prevalence, while in a clinical study the cut-off point could be lowered with the aim of optimizing sensitivity.

First we estimated a cut-off point with high SN (>0.80) and acceptable SP (>0.70), or failing this, the best Youden’s Index score which balances SN and SP result[48]. Subsequently, we estimated a cut-off point to optimize concordance on prevalence estimate between online survey test and MINI interview[49]. For a binary response, this is assessed with McNemar’s test, a modification of the ordinary chi-square test that takes the paired nature of the responses into account. A statistically significant result (p<0.05) shows that there is evidence of a systematic difference between the proportion of cases from the two tests. If one test is the gold-standard, the absence of a systematic difference implies that there is no bias on prevalence estimate[49]. Inherently, we created a dichotomization of screening scales to differentiate predicted cases from non-cases. As a result, we presented these analyses for 12-month and lifetime diagnoses.

## Results

### Participants

**Fig 1:**
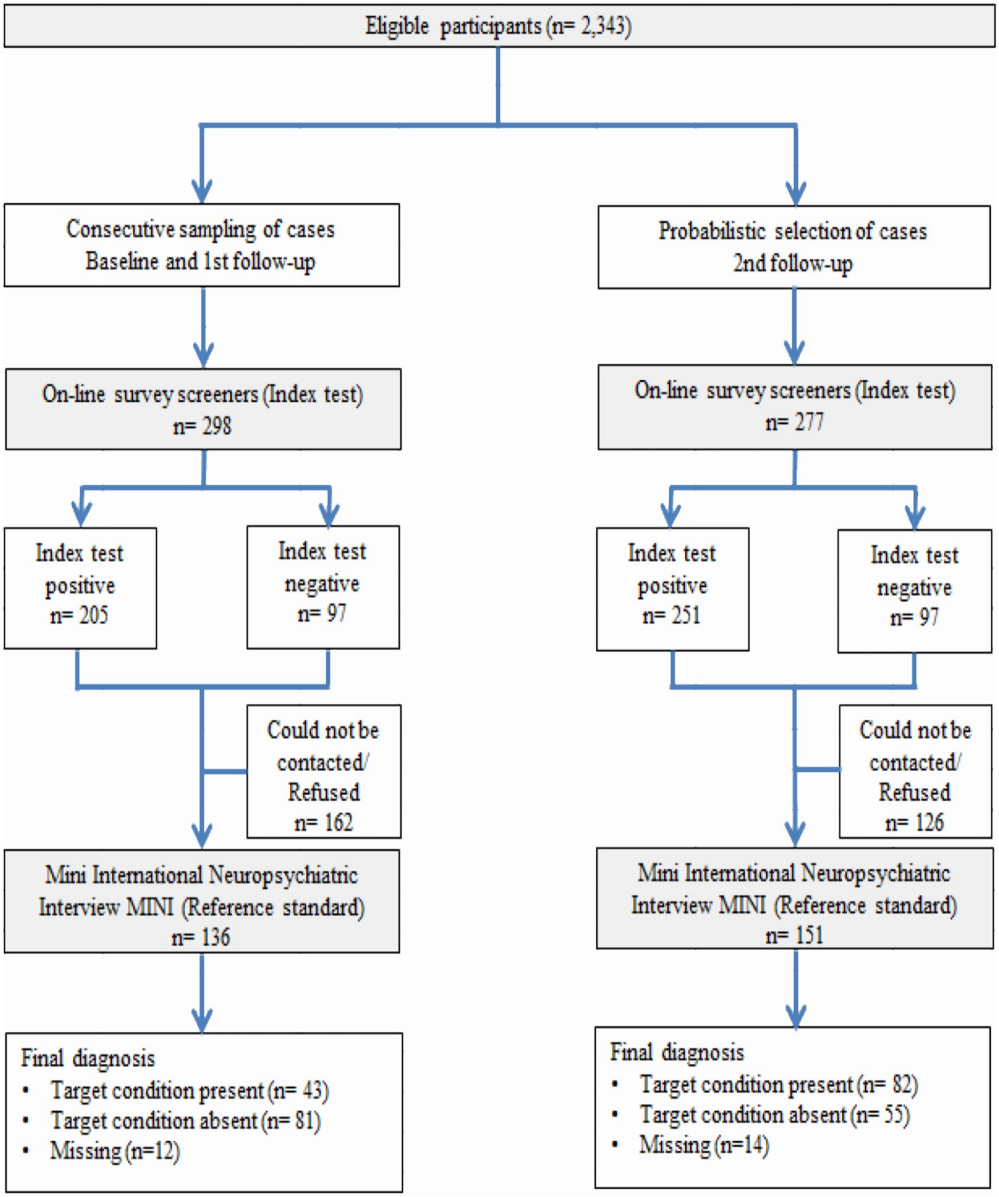
Modified version of STARD 2015 flow diagram of participants through clinical reappraisal study.

Between May 2015 and July 2017, 575students were assessed for initial eligibility and invited to participate in the clinical reappraisal. In total, 287 (49.9%) completed the reappraisal study (the other288 could not be contacted or refused the phone interview). Fig 1 shows the flow of included participants through the study.

Table 1 compares the overall UNIVERSAL sample and the clinical reappraisal subsample. The majority of the latter were female (n = 216), with ages 18 and 19 (n = 231), Spanish (n = 258) and came from Social (n = 108) and Health Sciences (n = 85) study fields. After weighting, the distribution of the reappraisal subsample was very similar to the overall UNIVERSAL sample, except for age. In the reappraisal sub-sample at baseline survey, mood disorders and anxiety disorders were more frequent than in the overall sample, both in the last 12-months and lifetime.12-month STB was 7.2% in the clinical reappraisal sub-sample and 9.2% in the overall sample, while STB lifetime in the reappraisal sub-sample was more frequent (21.8%) than in the overall sample (24.0%). There was significant difference in prevalence in the initial sample and the clinical reappraisal sample on anxiety disorders lifetime (p = 0.004) in spite of the use of post-stratification weights were used (Table 1).

**Table 1:** Characteristics of the *UNIVERSAL* baseline online survey on the overall sample and clinical reappraisal sub-sample (unweighted observations and weighted percentages).

		Overall sample n = 2,343		Clinical reappraisal sub-sample n = 287		
		n	%	n	%	*p-value*
Sex	Female	1,691	55.4	216	55.3	*0*.*97*
Age	18	1644	61.0	177	70.0	*0*.007*
	19	358	20.2	54	14.5	
	20+	341	18.7	56	15.5	
Nationality	Non-Spanish	128	2.9	29	2.9	*1*.*00*
Field of studies	Arts & Humanities	267	9.8	37	9.8	*1*.*00*
	Health Sciences	595	15.7	85	15.7	
	Social Sciences	947	47.6	108	47.6	
	Science	216	8.4	25	8.4	
	Engineering & Architecture	318	18.6	32	18.6	
Any mood^a^	12m	536	19.6	79	20.7	*0*.*64*
	Lifetime	644	23.8	100	25.9	*0*.*40*
Any anxiety^b^	12m	463	16.3	85	18.9	*0*.*23*
	Lifetime	547	20.1	104	26.9	*0*.004*
Suicidal thoughts and behaviors^c^	12m	238	9.2	37	7.2	*0*.*24*
	Lifetime	538	21.8	98	24.0	*0*.*37*

^a^.- Mood include: Major Depression Episode or Mania/Hypomania, assessed with the Composite International Diagnostic Interview Screening Scales [CIDI-SC];

^b^.- Anxiety include: Panic Disorder or Generalized Anxiety Disorder, assessed with the Composite International Diagnostic Interview Screening Scales [CIDI-SC];

^c^.- Suicidal thoughts and behaviors based on definition used in Spain suicide prevalence paper (Blasco MJ et al, *Suic*. *Suicide Life Threat Behav*. 2019) including: suicidal ideation, suicide plan and suicide attempt (excluding the questions of death wish and non-suicidal self-injury), assessed with questions from the Self-Injurious Thoughts and Behaviors Interview [SITBI] and the Columbia-Suicide Severity Rating Scale [C-SSRS].

*P-value statistically significant 0.05.

### Prevalence estimates of the MINI based on the WMH-ICS online survey screeners

Weighted prevalence estimates according to the online survey screeners and MINI showed statistically significant differences for most of the disorders (p<0.05), except for 12-months and lifetime M/H and PD **(**Table 2). The online screening scales showed a higher prevalence than the MINI estimates for mood disorders 12-month (15.4% vs. 7.3%) and lifetime (34.3% vs. 18.6%). However, prevalence disagreements varied across individual mood disorders, with statistical significant differences in 12-month and lifetime MDE (5.8% vs 13.7%; 16.5% vs. 32.9%, respectively); and not statistically significant differences on M/H prevalence. Disagreement in prevalence estimates were also found for 12-month and lifetime anxiety disorders (16.3 vs. 3.7%; 32.4% vs. 10.6%, respectively) but disagreements varied across individual disorders:12-month and lifetime GAD prevalence was higher for online survey screeners than for the MINI while the opposite was found for PD, although differences were not statistically significant. Prevalence estimates of WMH-ICS online survey screeners were higher than the MINI for 12-month and lifetime STB (8.5% vs. 5.0%; 25.7% vs.16.2%, respectively)(Table 2).

**Table 2:** Prevalence estimates of common mental disorders and suicidal thoughts and behaviors in the clinical reappraisal sample, according to the WMH-ICS online survey screeners and the MINI (n = 287) (unweighted observations and weighted percentages).

		Clinical Reappraisal sample							
		Online survey screeners^			MINI			McNemar	
		n	%	CI 95%	n	%	CI 95%	χ^2^	p-value
**Mental disorders**									
**Any mood**^a^	12-m	67	15.4	11.2–19.5	32	7.3	4.3–10.3	14.6	0.0001*
	Lifetime	136	34.3	28.8–39.8	66	18.6	14.1–23.1	22.7	<.0001*
Major depressive episode	12-m	60	13.7	9.8–17.7	26	5.8	3.1–8.5	15.9	<.0001*
	Lifetime	129	32.9	27.5–38.4	58	16.5	12.2–20.8	24.9	<.0001*
Mania/Hypomania	12-m	14	2.9	1.0–4.8	7	1.8	0.2–3.3	1.03	0.310
	Lifetime	25	4.9	2.4–7.4	13	3.0	1.1–5.0	1.52	0.218
**Any anxiety**^b^	12-m	70	16.3	12.0–20.6	19	3.7	1.5–5.9	29.8	<.0001*
	Lifetime	129	32.4	27.0–37.8	32	10.6	7.0–14.1	44.3	<.0001*
Panic disorder	12-m	5	1.3	0.0–2.6	17	3.3	1.2–5.4	3.35	0.067
	Lifetime	20	6.0	3.2–8.7	28	9.3	6.0–12.7	2.34	0.126
Generalized anxiety disorder	12-m	67	15.4	11.3–19.6	7	1.4	0.0–2.7	38.3	<.0001*
	Lifetime	123	30.7	25.4–36.1	11	2.5	0.7–4.4	78.2	<.0001*
**Suicidal thoughts and behaviors**									
Suicidal thoughts and behaviors^c^	12-m	35	8.5	5.3–11.8	25	5.0	2.5–7.5	6.29	0.012*
	Lifetime	104	25.7	20.7–30.8	71	16.2	11.9–20.5	17.9	<.0001*

^a^.- Mood include: Major Depression Episode or Mania/Hypomania, assessed with the Composite International Diagnostic Interview Screening Scales [CIDI-SC];

^b^.- Anxiety include: Panic Disorder or Generalized Anxiety Disorder, assessed with the Composite International Diagnostic Interview Screening Scales [CIDI-SC];

^c^.- Suicidal thoughts and behaviors based on definition used in Spain suicide prevalence paper (Blasco MJ et al, *Suic*. *Suicide Life Threat Behav*. 2019) including: suicidal ideation, suicide plan and suicide attempt (excluding the questions of death wish and non-suicidal self-injury), assessed with questions from the Self-Injurious Thoughts and Behaviors Interview [SITBI] and the Columbia-Suicide Severity Rating Scale [C-SSRS].

^Prevalence estimates according to the reappraisal temporary moment (see the Methods section)

*P-value statistically significant 0.05. CI95%: Confidence interval according 0.05 error.

### Operating Characteristics of WMH-ICS online survey screeners

In Table 3, the online screeners showed a SN in detecting mood disorders of 67.0% at 12-month and 65.0% lifetime. In the case of anxiety, corresponding values were 76.8%, and 59.6%. For specific mental disorders, SN for 12-month and lifetime MDE was 70.8% and 61.8%, respectively; for both 12-month and lifetime GAD, SN was 100.0%. SN for PD was lower than 20%, and for M/H, it was lower than 33.6%. Proportions of correctly detected of12-month and lifetime STB cases were 75.9%, and 87.2%, respectively. Proportions of online screener cases confirmed by the MINI (PPV) ranged from 8.4% to 55.1%.

**Table 3:** WMH-ICS online survey screeners operating characteristics for estimating reference standard (MINI) prevalence (n = 287) (weighted values).

			Positive operating characteristics					Negative operating characteristics					AUC
		Cut-point	SN	SE(SN)	PPV	SE(PPV)	LR+	SP	SE(SP)	NPV	SE(NPV)	LR-	
**Mental disorders**													
**Any mood**^a^	12-m	—	67.0	10.3	31.6	7.0	5.9	88.6	2.0	97.1	1.1	0.4	0.78
	Lifetime	—	65.0	6.7	35.6	5.0	2.4	73.3	3.0	90.2	2.2	0.5	0.69
Major depressive episode	12-m	15	70.8	11.0	29.6	7.3	6.8	89.6	1.9	98.0	0.9	0.3	0.80
	Lifetime	15	61.8	7.2	31.3	4.9	2.3	73.3	2.9	90.7	2.1	0.5	0.68
Mania/Hypomania	12-m	4	33.6	21.1	20.6	13.5	14.6	97.7	0.9	98.8	0.7	0.7	0.66
	Lifetime	4	20.9	13.5	12.9	8.9	4.6	95.5	1.2	97.5	1.0	0.8	0.58
**Any anxiety**^b^	12-m	—	76.8	13.4	18.1	5.7	5.7	86.5	2.1	99.0	0.7	0.3	0.82
	Lifetime	—	59.6	9.1	19.1	4.1	2.1	71.1	2.9	93.9	1.7	0.6	0.65
Panic disorder	12-m	2	19.0	12.4	47.5	25.0	27.1	99.3	0.5	97.3	1.0	0.8	0.59
	Lifetime	2	18.3	7.6	27.8	10.9	3.8	95.2	1.3	92.1	1.7	0.9	0.57
Generalized anxiety disorder	12-m	21	100.0	0.0	9.1	4.4	7.2	86.2	2.1	100.0	0.0	NA	0.93
	Lifetime	21	100.0	0.0	8.4	3.0	3.5	71.4	2.7	100.0	0.0	NA	0.86
**Suicidal thoughts and behaviors**													
Suicidal thoughts and behaviors^c^	12-m	—	75.9	11.9	43.3	10.1	14.6	94.8	1.4	98.7	0.7	0.3	0.85
	Lifetime	—	87.2	5.0	55.1	5.9	6.4	86.3	2.3	97.2	1.2	0.1	0.87

^a^.- Mood include: Major Depression Episode or Mania/Hypomania, assessed with the Composite International Diagnostic Interview Screening Scales [CIDI-SC];

^b^.- Anxiety include: Panic Disorder or Generalized Anxiety Disorder, assessed with the Composite International Diagnostic Interview Screening Scales [CIDI-SC];

^c^.- Suicidal thoughts and behaviors based on definition used in Spain suicide prevalence paper (Blasco MJ et al, *Suic*. *Suicide Life Threat Behav*. 2019) including: suicidal ideation, suicide plan and suicide attempt (excluding the questions of death wish and non-suicidal self-injury), assessed with questions from the Self-Injurious Thoughts and Behaviors Interview [SITBI] and the Columbia-Suicide Severity Rating Scale [C-SSRS].

SN: Sensitivity (% of MINI cases detected by the UNIVERSAL instrument); PPV: Positive Predictive Value (% of UNIVERSAL cases confirmed by the MINI);LR+: likelihood ratio positive (proportion of reference standard cases testing positive according to the index test divided by the proportion of non-cases testing positive in the index test); SP: Specificity (% of MINI non-cases classified as non-cases by the UNIVERSAL instrument); NPV: Negative Predictive Value (% of UNIVERSAL non-cases confirmed as non-cases by the MINI); LR-: likelihood ratio negative (proportion of reference standard cases testing negative divided by the proportion of non-cases testing negative in the index test); SE: Standard Error. AUC: area under the receiver operating characteristic curve.

The proportion of non-cases correctly classified (SP) ranged from 71.1% to 99.3% for all 12-month and lifetime disorders and the proportions of online screeners non-cases confirmed by the MINI (NPV) were 90.2%-100.0%. The highest relative proportions of screened positives versus screened negatives confirmed as cases by the MINI reappraisal (LR+) generated moderate changes in posttest probability for 12-month STB (14.6), 12-month PD (27.1), and 12-month M/H (14.6). On the other hand, LR- values were good for lifetime STB, whilst for all other LR- values, this ranged from 0.3 to 0.9(Table 3).

With the Area Under the ROC curve (AUC) we aimed to obtain a single numerical value for the overall diagnostic accuracy of the screen measures. Individual-level concordance was fair to substantial for all disorders, obtaining AUCs ranging from 0.7 to 0.9, except slightly lower for lifetime M/H and for 12-month and lifetime PD (just below 0.6) (Table 3).

### Improving diagnostic capacity through cut-off point changes

In order to improve diagnostic capacity for MDE, M/H, PD and GAD, we carried out a sensitivity analysis according to two different criteria to select optimal cut-off points for each diagnostic: a) maximization of SN; or b) optimization of concordance on prevalence. Table 4 shows operating characteristics for estimating lifetime disorder. When SN was prioritized, an increase of the online survey lifetime prevalence estimate was found for all disorders other than GAD, which presented a lower prevalence in comparison to the initial algorithms. This difference was due to the fact that GAD originally had SN = 100% and when a better balance between SN and SP was achieved, its prevalence estimate decreased slightly, obtaining a SN = 97.3% lifetime. PPVs were higher than the original algorithms, ranging from 10.5 to 36.6. SP decreased slightly in comparison to original algorithms ranging from 59.7% to 83.2% for all disorders, but NPV increased ranging from 96.7 to 99.9. LR+ values for all disorders were higher than the original algorithms and LR- values ranged from 0.1 to 0.3. For mood disorders and anxiety disorders, the AUC increased slightly in comparison to the initial algorithm (from fair to substantial). For M/H and PD the increase in AUC was somewhat higher (from slight to moderate or substantial) (Table 4).

**Table 4:** WMH-ICS online survey screeners operating characteristics for estimating reference standard (MINI) lifetime prevalence when diagnostic cut-off points are changed to maximize sensitivity or have an optimal prevalence (n = 287) (weighted values).

	Cut-point	Online survey screeners prevalence estimate^^^		Positive operating characteristics					Negative operating characteristics					McNemar		AUC
		%	SE(%)	SN	SE(SN)	PPV	SE(PPV)	LR+	SP	SE(SP)	NPV	SE(NPV)	LR-	χ^2^	p-value	
**Mental disorders**																
**Any mood**^a^																
High SN	--	51.0	2.9	94.8	3.1	35.1	4.0	2.4	59.7	3.3	98.1	1.2	0.1	82.62	<.0001*	0.77
Optimal for prevalence	--	23.2	2.5	52.8	7.0	42.7	6.2	3.3	84	2.5	88.7	2.2	0.6	2.38	0.123	0.68
Major depressive episode																
High SN	14	40.1	2.9	88.9	4.7	36.6	4.6	2.9	69.8	3.0	97.0	1.3	0.2	56.0	<.0001*	0.79
Optimal for prevalence	17	21.6	2.4	48.6	7.5	37.3	6.3	3.0	84.0	2.4	89.3	2.1	0.6	3.09	0.079	0.66
Mania/hypomania																
High SN	2	24.4	2.5	83.7	13.1	10.5	3.7	3.7	77.6	2.5	99.3	0.6	0.2	57.2	<.0001*	0.81
Optimal for prevalence	4	4.9	1.3	20.9	13.5	12.9	8.9	4.6	95.5	1.2	97.5	1.0	0.8	1.52	0.218	0.58
**Any anxiety**^b^																
High SN	--	35.7	2.8	91.9	5.1	26.5	4.4	3.1	70.7	2.9	98.7	0.8	0.1	67.02	<.0001*	0.81
Optimal for prevalence	--	9.9	1.8	29.4	8.6	29.9	8.6	3.7	92.1	1.7	91.9	1.7	0.8	0.01	0.942	0.61
Panic disorder																
High SN	3	21.7	2.4	71.1	9.1	29.6	5.8	4.2	83.2	2.3	96.7	1.2	0.3	25.7	<.0001*	0.77
Optimal for prevalence	5	7.0	1.5	19.8	7.8	25.6	9.8	3.5	94.3	1.4	92.2	1.6	0.9	0.96	0.328	0.57
Generalized anxiety disorder																
High SN	24	22.7	2.5	97.3	6.1	11.0	3.9	4.7	79.4	2.4	99.9	0.2	0.0	55.6	<.0001*	0.88
Optimal for prevalence	31	4.0	1.2	52.6	18.9	32.8	13.6	18.8	97.2	1.0	98.7	0.7	0.5	1.67	0.196	0.75
**Suicidal thoughts and behaviors**																
Suicidal thoughts and behaviors^c^^^	--	25.7	2.6	87.2	5.0	55.1	5.9	6.4	86.3	2.3	97.2	1.2	0.1	17.9	<.0001*	0.85

^a^.- Mood include: Major Depression Episode or Mania/Hypomania, assessed with the Composite International Diagnostic Interview Screening Scales [CIDI-SC];

^b^.- Anxiety include: Panic Disorder or Generalized Anxiety Disorder, assessed with the Composite International Diagnostic Interview Screening Scales [CIDI-SC];

^c^.- Suicidal thoughts and behaviors based on definition used in Spain suicide prevalence paper (Blasco MJ et al, *Suic*. *Suicide Life Threat Behav*. 2019) including: suicidal ideation, suicide plan and suicide attempt (excluding the questions of death wish and non-suicidal self-injury), assessed with questions from the Self-Injurious Thoughts and Behaviors Interview [SITBI] and the Columbia-Suicide Severity Rating Scale [C-SSRS].

^Prevalence estimates according to the reappraisal temporary moment (see the Methods section).

^^ Suicidal thoughts and behaviors diagnostic algorithm is created according Spanish suicide definition, without cut-off points.

*P-value statistically significant 0.05.

SN: Sensitivity (% of MINI cases detected by the UNIVERSAL instrument); PPV: Positive Predictive Value (% of UNIVERSAL cases confirmed by the MINI);LR+: likelihood ratio positive (proportion of reference standard cases testing positive according to the index test divided by the proportion of non-cases testing positive in the index test); SP: Specificity (% of MINI non-cases classified as non-cases by the UNIVERSAL instrument); NPV: Negative Predictive Value (% of UNIVERSAL non-cases confirmed as non-cases by the MINI); LR-: likelihood ratio negative (proportion of reference standard cases testing negative divided by the proportion of non-cases testing negative in the index test); SE: Standard Error; AUC: area under the receiver operating characteristic curve.

Table 4 also shows the implications of making changes in the cut-off points to obtain comparable prevalence estimates. Special cases were M/H and PD, for which no statistical significant differences were found in prevalence estimates using initial algorithms. Both algorithms could be enhanced by changing cut-off points, but their operating characteristics did not get better. Compared to the original algorithms, prevalence estimates were decreased, getting closer to that of the reference measure, at the expense of a lower SN and AUC for overall mood and anxiety diagnoses. PPVs slightly increase regarding to the original algorithm with a range 12.9–42.7 and NPVs were 88.7–98.7.

Table 5 shows operating characteristics for estimating 12-month prevalence when cut-off points were changed. Results in the same direction than Table 4 were found, improving SN in all disorders when SN was maximized. Even though, when the cut-off point was the optimal for prevalence, statistical significant differences were found in mood disorders, MDE and STB prevalence.

**Table 5:** WMH-ICS online survey screeners operating characteristics for estimating reference standard (MINI) 12-month prevalence when diagnostic cut-off points are changed to maximize sensitivity or have an optimal prevalence (n = 287) (weighted values).

	Cut-point	Online survey screeners prevalence estimate^^^		Positive operating characteristics					Negative operating characteristics					McNemar		AUC
		%	SE(%)	SN	SE(SN)	PPV	SE(PPV)	LR+	SP	SE(SP)	NPV	SE(NPV)	LR-	χ^2^	p-value	
**Mental disorders**																
**Any mood**^a^																
High SN	--	23.9	2.5	76.1	9.3	22.9	5.1	3.8	79.8	2.5	97.7	1.0	0.3	39.58	<.0001*	0.78
Optimal for prevalence	--	11.9	1.9	50.5	11.2	30.4	7.9	5.5	90.9	1.8	95.9	1.3	0.5	5.44	0.019*	0.71
Major depressive episode																
High SN	14	16.0	2.2	73.0	11.1	26.2	6.5	5.8	87.4	2.0	98.1	0.9	0.3	22.4	<.0001*	0.80
Optimal for prevalence	17	10.0	1.8	49.8	12.5	28.4	8.4	6.5	92.3	1.6	96.8	1.1	0.5	5.30	0.021*	0.71
Mania/hypomania																
High SN	2	11.2	1.9	33.6	21.1	5.3	4.0	3.1	89.2	1.9	98.7	0.7	0.7	21.7	<.0001*	0.61
Optimal for prevalence	4	2.9	1	33.6	21.1	20.6	13.5	14.6	97.7	0.9	98.8	0.7	0.7	1.03	0.310	0.66
**Any anxiety**^b^																
High SN	--	13.8	2.0	78.7	12.9	21.7	6.7	7.2	89	1.9	99.1	0.6	0.2	23.82	<.0001*	0.84
Optimal for prevalence	--	3.6	1.1	39.2	15.4	40.2	15.5	17	97.7	0.9	97.7	0.9	0.6	0.01	0.941	0.68
Panic disorder																
High SN	3	3.7	1.1	44.8	16.6	39.7	15.5	19.5	97.7	0.9	98.1	0.8	0.6	0.12	0.725	0.71
Optimal for prevalence	5	1.8	0.8	17.8	12.1	32.0	19.0	13.7	98.7	0.7	97.2	1.0	0.8	1.55	0.213	0.58
Generalized anxiety disorder																
High SN	24	11.7	1.9	95.0	10.9	11.3	5.5	9.2	89.7	1.8	99.9	0.2	0.1	28.1	<.0001*	0.92
Optimal for prevalence	31	1.9	0.8	41.0	24.6	29.0	18.5	29.3	98.6	0.7	99.2	0.5	0.6	0.42	0.517	0.70
**Suicidal thoughts and behaviors**																
Suicidal thoughts and behaviors^c^	--	8.5	1.6	75.9	11.9	43.3	10.1	14.6	94.8	1.4	98.7	0.7	0.3	6.29	0.012*	0.81

^a^.- Mood include: Major Depression Episode or Mania/Hypomania, assessed with the Composite International Diagnostic Interview Screening Scales [CIDI-SC];

^b^.- Anxiety include: Panic Disorder or Generalized Anxiety Disorder, assessed with the Composite International Diagnostic Interview Screening Scales [CIDI-SC];

^c^.- Suicidal thoughts and behaviors based on definition used in Spain suicide prevalence paper (Blasco MJ et al, *Suic*. *Suicide Life Threat Behav*. 2019) including: suicidal ideation, suicide plan and suicide attempt (excluding the questions of death wish and non-suicidal self-injury), assessed with questions from the Self-Injurious Thoughts and Behaviors Interview [SITBI] and the Columbia-Suicide Severity Rating Scale [C-SSRS].

^Prevalence estimates according to the reappraisal temporary moment (see the Methods section).

^^ Suicidal thoughts and behaviors diagnostic algorithm is created according Spanish suicide definition, without cut-off points.

*P-value statistically significant 0.05.

SN: Sensitivity (% of MINI cases detected by the UNIVERSAL instrument); PPV: Positive Predictive Value (% of UNIVERSAL cases confirmed by the MINI);LR+: likelihood ratio positive (proportion of reference standard cases testing positive according to the index test divided by the proportion of non-cases testing positive in the index test); SP: Specificity (% of MINI non-cases classified as non-cases by the UNIVERSAL instrument); NPV: Negative Predictive Value (% of UNIVERSAL non-cases confirmed as non-cases by the MINI); LR-: likelihood ratio negative (proportion of reference standard cases testing negative divided by the proportion of non-cases testing negative in the index test); SE: Standard Error; AUC: area under the receiver operating characteristic curve.

The sensitivity, specificity, likelihood ratio positive (LR+), likelihood ratio negative (LR-), McNemar and Area Under the Curve (AUC) of different cut-off points for MDE, M/H, PD and GAD for estimating reference standard (MINI) lifetime and 12-month prevalence are shown in S1–S8 Tables.

## Discussion

This study evaluated the diagnostic concordance of online screener versions of the CIDI-SC, SITBI and C-SSRS with the MINI among Spanish university students. Overall concordance was reasonably adequate, particularly for 12-month and lifetime STB, showing optimal operating characteristics and substantial to almost perfect AUC. For 12-month Major Depressive Episode and Generalized Anxiety Disorder, online screener showed good SN, SP and NPVs with substantial AUC; however, Mania/Hypomania and Panic Disorder results were suboptimal. Overall diagnoses showed low PPVs both in the pre-specified cut-offs and the modified cut-offs. Thus, our findings regarding diagnostic accuracy should be interpreted with some caution.

### Comparison with previous studies

In general, results presented here are comparable to those found in previous research of the CIDI-SC—which have shown a good concordance with clinical diagnoses of mood and anxiety disorders [13,28,50,51]—and those of the SITBI and C-SSRS [30,34]. However, we found that individual-level concordance of mental disorders was somewhat lower than in previous psychometric studies of these scales. For the most part, our study found fair to moderate estimates (AUC = 0.60–0.79), whereas most previous evaluations found moderate to substantial concordance (AUC = 0.70–0.89).

The samples in most previous studies were different than university students, including Army personnel [13], primary care patients[28], and general population respondents [30,50]. Also, these studies validated mental disorder screening instruments that are not in conjunction with a suicidal thoughts and behaviors screening instrument, as it is in our study. Our results emphasize the need to carefully consider the characteristics of the population in which there is a desire to use a screening instrument [52,53]. Furthermore, we used screening scales diagnostic algorithms from the ARMY STARRS survey and we adapted them for use in the WMH-ICS self-administered questionnaire[13]. In fact, differences between our sample and that of the previous study could modify the operating characteristics of the online survey screeners. For this reason, we carried out this study to investigate the extent to which the screening scales’ diagnostic algorithms were valid and applicable in a sample of different characteristics to the military sample.

Web-based questionnaires have become an important tool in epidemiologic data collection, especially for recruitment and follow-up of large cohorts[54], even though they have often not been validated specifically for the assessment of mental disorders in university populations. Several programs through which people may be assessed for mental disorders through the Internet have evaluated the validity of a web-based instrument for common mental disorders in the general population or in clinical samples [21–23,54]. The WMH-ICS online screening scales showed similar SN, SP and NPVs values to other web-based screening instruments for mental disorders [21–23] (SN:71.0–1.00; SP:73.0–97.0; NPVs:85.0–1.00), when we adjusted the cut-off points according to SN. However, our study showed low PPVs for both the initial algorithms and after obtaining modified cut-offs. Similar low values were also reported in another study (11.0–51.0)[21], whose authors argue that they might be due to a low prevalence of some of the mental disorders assessed. Other studies that validated self-administered instruments showed similarly modest psychometric properties for SN (range from 72.2–92.0) but found higher PPVs (range from 40.0 to 87.0) than our study. Nonetheless, and in contrast with our results, these studies showed also low values for SP and NPVs (SP: 29.0–78.0; NPVs: 38.0–93.0).

College years period is well-known as a peak period to develop first onset on mental disorders[3,4]. Our results provide evidence of validity of online screener measures among this population, and they might be instrumental to facilitate the implementation of health programs to diminish the impact of mental disorders in this crucial period [3–7]. Further, there is potential to facilitate web-based interventions, which may be valuable to improve student mental health [55–57]. Indeed, the epidemiological surveys in the university context can be the first step to implement state-of-the-art web-based interventions about health promotion and prevention of mental disorders among university students.

### Modification of WMH-ICS online survey screeners’ cut-off points

Definitions of screened positives and screened negatives could be enhanced by selecting the cut-off point that optimizes the test performance indicators that are deemed useful at each specific research objective. Different applications, like epidemiological as well as clinical, might use screening instruments for different purposes and depending on them, the cut-off point decision can be changed [13]. The accuracy of a diagnostic index test is not constant but varies across different clinical contexts, disease spectrums and even patient subgroups [58]. In a clinical study, screening instruments might be used to select people for treatment more in-depth or invasive diagnosis assessment, and it can be more relevant to achieve high sensitivity to capture real cases by the screening instruments [13,28,40].

We, therefore, investigated whether increasing the cut-off point could reach at a minimum SN of 80% (or the best balance between SN and SP) with the result that most MINI cases would be correctly identified by the online survey. However, we observed low PPVs and research to further improve diagnostic algorithms of these online screeners for clinical purposes is necessary.

Nonetheless, for epidemiological research, it may be important to obtain unbiased estimates of the prevalence of the disorder to assess distribution of mental disorders in the university population through an online survey[13,59]. This approximation would allow to monitor prevalence trends of mental disorders and to evaluate interventions in the university population. Choosing a lower cut-off point would provide a higher concordance on the prevalence estimates based on McNemar test. Other ways to improve diagnostic capacity implies PPV and NPV. However, the predictive values of a study can not be generalized due to the relationship with the prevalence of the disease[60].

### Limitations

Several limitations of the study should be taken into consideration when interpreting our results. First, we used the MINI as the “gold standard” diagnostic instrument which might be considered a sub-optimal standard, in particular since it was administered via telephone by more than one psychologist, and it provides a brief content about diagnoses. We nevertheless implemented the MINI for feasibility and because it has shown to have a SN/SP above the minimum acceptable level (.8/.8) with structured interviews [61]. The MINI interview has been used widely in clinical context as well as in the research context. Several studies showed good psychometric properties what could define it as valid “gold standard” [15,61,62]. However, a risk of bias towards positive results has been reported and conducting the MINI after the CIDI could induce a “learning” bias. Nonetheless, the short duration of the MINI may have helped to prevent participants’ negative answers to reduce the interview duration [63]. Also, previous research shows that respondents in community surveys tend to report less as they are interviewed more due to respondent fatigue, as a result lower bound estimates of concordance [64]. Given that, the second interview was blinded for interviewers and respondents. In spite of this would have decreased concordance, our concordance results are almost high. Besides, face-to-face interviews are typically enriched with non-verbal information which may increase diagnostic validity, while we administered the MINI by phone. Nevertheless, research shows that telephone vs in-person modes seem not to influence findings [39,65,66]. In addition, all interviewers were clinical psychologists with experience in the use of the MINI and they had a learning session to maximize the similarity in data collection. Finally, in our study inter-rater reliability was not assessed and therefore we do not know the reproducibility of our study. This reinforces the need to interpret the results cautiously. Further research should estimate inter-rater reliability and test-retest analyses.

Second, although unlikely, it is possible that an undetected disorder in the online survey appeared in the time before the clinical reappraisal. Also, it is possible that the period for an existing disorder at the time of the online evaluation had expired at the time of the reappraisal. We combatted these risks by allowing a maximum of 4 weeks between online and reappraisal evaluations [67], while in other studies recall periods were shorter from the same session to two weeks[13,50,63]. However, disease progression bias are more likely to have significant effects on studies of tests for acute diseases (i.e., infections) that may change more rapidly [68]. Third, current results are based on a relatively small number of cases for some of the mental disorders considered. This is especially true for M/H and PD, with the lowest prevalence and showed poor accuracy. An important task for future studies will be to estimate their accuracy in larger samples, which, at the same time, would allow for subgroup analyses. Fourth, to assure sufficient number of individuals for each disorder studied, we carried out a probabilistic selection of participants in the reappraisal study. We performed weighted analyses that restored the distribution of disorders in the student population, which assures unbiased estimates. Fifth, students could show different levels of trust and confidence to the clinical reappraisal in comparison to a more confidential evaluation as the online survey. Social desirability bias occurs often when a person answers according to the expectation of the other [69]. The degree this might have contributed to a lower prevalence of disorders in the reappraisal assessment and that to the assessment of validity of the screeners remains to be studied.

Finally, we calculated AUC from ROC curves for each dichotomous screening scale. However, dichotomization often discards potentially useful information that would be retained with the interpretation of scores along the continuum of the disease[70]. Therefore future research should address the accuracy of these online survey screeners as a continuous measure that allows valuable information of different severity levels.

## Conclusions

Our findings suggest that while the screening scales used in the UNIVERSAL online survey tend to overestimate true diagnostic prevalence, they are nonetheless valuable in making it possible to screen quickly and efficiently for common mental disorders in a way that captures that large majority of true cases. This is especially true for 12-month prevalence disorders, where the instrument showed better diagnostic capacity. Since the post hoc derivation of a diagnostic threshold can introduce a bias into diagnostic test validity, it is necessary replicate these analyses in other countries which use WMH-ICS initiative. Such replication should explore to what extent predictive values from one study should transferred to another setting with a different prevalence of the disease in the population [28].

## Supporting information

S1 Table Sensitivity, specificity, likelihood ratio positive (LR+), likelihood ratio negative (LR-), McNemar and Area Under the Curve (AUC) for different cut-off points of Major Depressive Episode 12-month algorithm for estimating reference standard (MINI)(n = 287). (PDF)

S2 Table Sensitivity, specificity, likelihood ratio positive (LR+), likelihood ratio negative (LR-), McNemar and Area Under the Curve (AUC) for different cut-off points of Major Depressive Episode lifetime algorithm for estimating reference standard (MINI)(n = 287). (PDF)

S3 Table Sensitivity, specificity, likelihood ratio positive (LR+), likelihood ratio negative (LR-), McNemar and Area Under the Curve (AUC) for different cut-off points of Mania/Hypomania 12-month algorithm for estimating reference standard (MINI)(weighted values). (PDF)

S4 Table Sensitivity, specificity, likelihood ratio positive (LR+), likelihood ratio negative (LR-), McNemar and Area Under the Curve (AUC) for different cut-off points of Mania/Hypomania lifetime algorithm for estimating reference standard (MINI)(weighted values). (PDF)

S5 Table Sensitivity, specificity, likelihood ratio positive (LR+), likelihood ratio negative (LR-), McNemar and Area Under the Curve (AUC) for different cut-off points of Panic Disorder 12-month algorithm for estimating reference standard (MINI)(n = 287). (PDF)

S6 Table Sensitivity, specificity, likelihood ratio positive (LR+), likelihood ratio negative (LR-), McNemar and Area Under the Curve (AUC) for different cut-off points of Panic Disorder lifetime algorithm for estimating reference standard (MINI)(n = 287). (PDF)

S7 Table Sensitivity, specificity, likelihood ratio positive (LR+), likelihood ratio negative (LR-), McNemar and Area Under the Curve (AUC) for different cut-off points of Generalized Anxiety Disorder 12-month algorithm for estimating reference standard (MINI)(n = 287). (PDF)

S8 Table Sensitivity, specificity, likelihood ratio positive (LR+), likelihood ratio negative (LR-), McNemar and Area Under the Curve (AUC) for different cut-off points of Generalized Anxiety Disorder lifetime algorithmfor estimating reference standard (MINI) (n = 287). (PDF)

S9 Table Prevalence estimates of common mental disorders and suicidal thoughts and behaviors in the clinical reappraisal samples recruited at each follow-up, according to the online survey screeners and the MINI (n = 287) (unweighted values). (PDF)

S1 STARD Checklist (PDF)
