# Supplementary material for: Accuracy of online survey assessment of mental disorders and suicidal thoughts and behaviors in Spanish university students. Results of the WHO World Mental Health- International College Student initiative
Source: PLoS One. 2019 Sep 5;14(9):e0221529. doi: 10.1371/journal.pone.0221529 (PMC6728025; doi:10.1371/journal.pone.0221529)
Supplement: S1 Table — (PDF) [file pone.0221529.s001.pdf]

S1 Table. Sensitivity, specificity, likelihood ratio positive (LR+), likelihood ratio negative (LR-), McNemar and Area Under the Curve (AUC) for different cut-off points of Major Depressive Episode 12-month algorithm for estimating reference standard (MINI) (n=287)

Cutpoint	Sensitivity	Specificity	LR+	LR-	McNemar		AUC
					χ2	p-value	
(>= 1)	97.8	64.6	2.8	0	92.8	<.0001*	0.81
(>= 2)	97.8	64.6	2.8	0	92.8	<.0001*	0.81
(>= 3)	97.8	64.6	2.8	0	92.8	<.0001*	0.81
(>= 4)	97.8	64.6	2.8	0	92.8	<.0001*	0.81
(>= 5)	97.8	65.0	2.8	0	91.7	<.0001*	0.81
(>= 6)	97.8	65.0	2.8	0	91.7	<.0001*	0.81
(>= 7)	97.8	65.0	2.8	0	91.7	<.0001*	0.81
(>= 8)	97.8	65.5	2.8	0	90.4	<.0001*	0.82
(>= 9)	97.8	67.2	3.0	0	86.1	<.0001*	0.83
(>= 10)	97.8	67.2	3.0	0	86.1	<.0001*	0.83
(>= 11)	92.5	69.6	3.0	0.1	77.1	<.0001*	0.81
(>= 12)	92.5	71.7	3.3	0.1	71.5	<.0001*	0.82
(>= 13)	92.5	82.8	5.4	0.1	42.3	<.0001*	0.88
(>= 14)	73.0	87.4	5.8	0.3	22.4	<.0001*	0.80
(>= 15)	70.8	89.6	6.8	0.3	15.9	<.0001*	0.80
(>= 16)	60.3	91.5	7.1	0.4	8.84	0.003*	0.76
(>= 17)	49.8	92.3	6.5	0.5	5.3	0.021*	0.71
(>= 18)	46.9	93.9	7.7	0.6	2.28	0.131	0.70
(>= 19)	43.7	96.9	14.1	0.6	0.04	0.835	0.70
(>= 20)	26.4	97.2	9.4	0.8	1.15	0.283	0.62
(>= 21)	16.1	97.4	6.2	0.9	2.22	0.136	0.57
(>= 22)	5.60	98.0	2.8	1	4.79	0.029*	0.52
(>= 23)	5.60	98.7	4.3	1	7.81	0.005*	0.52
(>= 24)	2.90	99.0	2.9	1	9.53	0.002*	0.51

*P-value statistically significant 0.05.
