# Supplementary material for: Accuracy of online survey assessment of mental disorders and suicidal thoughts and behaviors in Spanish university students. Results of the WHO World Mental Health- International College Student initiative
Source: PLoS One. 2019 Sep 5;14(9):e0221529. doi: 10.1371/journal.pone.0221529 (PMC6728025; doi:10.1371/journal.pone.0221529)
Supplement: S2 Table — (PDF) [file pone.0221529.s002.pdf]

S2 Table. Sensitivity, specificity, likelihood ratio positive (LR+), likelihood ratio negative (LR-), McNemar and Area Under the Curve (AUC) for different cut-off points of Major Depressive Episode lifetime algorithm for estimating reference standard (MINI) (n=287)

Cutpoint	Sensitivity	Specificity	LR+	LR-	McNemar		AUC
					χ^2	p-value	
(>= 1)	99	46.4	1.8	0	122.9	<.0001*	0.73
(>= 2)	99	46.4	1.8	0	122.9	<.0001*	0.73
(>= 3)	99	46.4	1.8	0	122.9	<.0001*	0.73
(>= 4)	99	46.4	1.8	0	122.9	<.0001*	0.73
(>= 5)	99	46.4	1.8	0	122.9	<.0001*	0.73
(>= 6)	99	46.4	1.8	0	122.9	<.0001*	0.73
(>= 7)	99	46.4	1.8	0	122.9	<.0001*	0.73
(>= 8)	99	47.5	1.9	0	120.3	<.0001*	0.73
(>= 9)	99	49.0	1.9	0	116.8	<.0001*	0.74
(>= 10)	97.1	50.1	1.9	0.1	111.1	<.0001*	0.74
(>= 11)	97.1	54.4	2.1	0.1	101.3	<.0001*	0.76
(>= 12)	96.3	57.7	2.3	0.1	92.8	<.0001*	0.77
(>= 13)	95.9	64.9	2.7	0.1	75.5	<.0001*	0.80
(>= 14)	88.9	69.8	2.9	0.2	56.0	<.0001*	0.79
(>= 15)	61.8	73.3	2.3	0.5	24.9	<.0001*	0.68
(>= 16)	53.5	78.1	2.4	0.6	12.2	0.001*	0.66
(>= 17)	48.6	84.0	3.0	0.6	3.09	0.078	0.66
(>= 18)	46.6	86.6	3.5	0.6	0.82	0.365	0.67
(>= 19)	43.5	91.6	5.2	0.6	0.89	0.346	0.68
(>= 20)	33.6	92.5	4.5	0.7	3.43	0.064	0.63
(>= 21)	25.0	94.4	4.5	0.8	9.09	0.003*	0.60
(>= 22)	15.6	95.1	3.2	0.9	14.1	0.0002*	0.55
(>= 23)	12.1	95.9	3.0	0.9	17.9	<.0001*	0.54
(>= 24)	6.60	98.5	4.4	0.9	32.2	<.0001*	0.53

*P-value statistically significant 0.05.
