# Supplementary material for: Accuracy of online survey assessment of mental disorders and suicidal thoughts and behaviors in Spanish university students. Results of the WHO World Mental Health- International College Student initiative
Source: PLoS One. 2019 Sep 5;14(9):e0221529. doi: 10.1371/journal.pone.0221529 (PMC6728025; doi:10.1371/journal.pone.0221529)
Supplement: S3 Table — (PDF) [file pone.0221529.s003.pdf]

S3 Table. Sensitivity, specificity, likelihood ratio positive (LR+), likelihood ratio negative (LR-), McNemar and Area Under the Curve (AUC) for different cut-off points of Mania/Hypomania 12-month algorithm for estimating reference standard (MINI) (weighted values)

Cutpoint	Sensitivity	Specificity	LR+	LR-	McNemar		AUC
					χ^2	p-value	
(>= 2)	33.6	89.2	3.1	0.7	21.7	<.0001*	0.61
(>= 3)	33.6	91.9	4.1	0.7	14.4	0.0001*	0.63
(>= 4)	33.6	97.7	14.6	0.7	1.03	0.310	0.66

*P-value statistically significant 0.05.
