# Supplementary material for: Accuracy of online survey assessment of mental disorders and suicidal thoughts and behaviors in Spanish university students. Results of the WHO World Mental Health- International College Student initiative
Source: PLoS One. 2019 Sep 5;14(9):e0221529. doi: 10.1371/journal.pone.0221529 (PMC6728025; doi:10.1371/journal.pone.0221529)
Supplement: S4 Table — (PDF) [file pone.0221529.s004.pdf]

S4 Table. Sensitivity, specificity, likelihood ratio positive (LR+), likelihood ratio negative (LR-), McNemar and Area Under the Curve (AUC) for different cut-off points of Mania/Hypomania lifetime algorithm for estimating reference standard (MINI) (weighted values)

Cutpoint	Sensitivity	Specificity	LR+	LR-	McNemar		AUC
					χ^2	p-value	
(>= 2)	83.7	77.6	3.7	0.2	57.2	<.0001*	0.81
(>= 3)	63.7	84.3	4.1	0.4	34.4	<.0001*	0.74
(>= 4)	20.9	95.5	4.6	0.8	1.52	0.218	0.58

*P-value statistically significant 0.05.
