# Supplementary material for: Accuracy of online survey assessment of mental disorders and suicidal thoughts and behaviors in Spanish university students. Results of the WHO World Mental Health- International College Student initiative
Source: PLoS One. 2019 Sep 5;14(9):e0221529. doi: 10.1371/journal.pone.0221529 (PMC6728025; doi:10.1371/journal.pone.0221529)
Supplement: S5 Table — (PDF) [file pone.0221529.s005.pdf]

S5 Table. Sensitivity, specificity, likelihood ratio positive (LR+), likelihood ratio negative (LR-), McNemar and Area Under the Curve (AUC) for different cut-off points of Panic Disorder 12-month algorithm for estimating reference standard (MINI) (n=287)

Cutpoint	Sensitivity	Specificity	LR+	LR-	McNemar		AUC
					χ^2	p-value	
(>= 1)	44.8	97.7	19.5	0.6	0.12	0.725	0.71
(>= 2)	44.8	97.7	19.5	0.6	0.12	0.725	0.71
(>= 3)	44.8	97.7	19.5	0.6	0.12	0.725	0.71
(>= 4)	39.8	98.2	22.1	0.6	0.06	0.809	0.69
(>= 5)	17.8	98.7	13.7	0.8	1.55	0.213	0.58
(>= 6)	17.8	99.6	44.5	0.8	5.19	0.023*	0.59
(>= 7)	4.90	100	NA	1	8.74	0.003*	0.52

*P-value statistically significant 0.05.
