# Supplementary material for: Accuracy of online survey assessment of mental disorders and suicidal thoughts and behaviors in Spanish university students. Results of the WHO World Mental Health- International College Student initiative
Source: PLoS One. 2019 Sep 5;14(9):e0221529. doi: 10.1371/journal.pone.0221529 (PMC6728025; doi:10.1371/journal.pone.0221529)
Supplement: S6 Table — (PDF) [file pone.0221529.s006.pdf]

S6 Table. Sensitivity, specificity, likelihood ratio positive (LR+), likelihood ratio negative (LR-), McNemar and Area Under the Curve (AUC) for different cut-off points of Panic Disorder lifetime algorithm for estimating reference standard (MINI) (n=287)

Cutpoint	Sensitivity	Specificity	LR+	LR-	McNemar		AUC
					χ^2	p-value	
(>= 1)	71.1	82.8	4.1	0.3	26.7	<.0001*	0.77
(>= 2)	71.1	82.8	4.1	0.3	26.7	<.0001*	0.77
(>= 3)	71.1	83.2	4.2	0.3	25.7	<.0001*	0.77
(>= 4)	36.1	87.6	2.9	0.7	5.09	0.024*	0.62
(>= 5)	19.8	94.3	3.5	0.9	0.96	0.328	0.57
(>= 6)	6.5	98.4	4.1	1	14.1	0.0002*	0.52
(>= 7)	3.2	99.6	8.0	1	21.9	<.0001*	0.51

*P-value statistically significant 0.05.
