# Supplementary material for: Accuracy of online survey assessment of mental disorders and suicidal thoughts and behaviors in Spanish university students. Results of the WHO World Mental Health- International College Student initiative
Source: PLoS One. 2019 Sep 5;14(9):e0221529. doi: 10.1371/journal.pone.0221529 (PMC6728025; doi:10.1371/journal.pone.0221529)
Supplement: S7 Table — (PDF) [file pone.0221529.s007.pdf]

S7 Table. Sensitivity, specificity, likelihood ratio positive (LR+), likelihood ratio negative (LR-), McNemar and Area Under the Curve (AUC) for different cut-off points of Generalized Anxiety Disorder 12-month algorithm for estimating reference standard (MINI) (n=287)

Cutpoint	Sensitivity	Specificity	LR+	LR-	McNemar		AUC
					χ^2	p-value	
(>= 13)	100	81.1	5.3	0	52.5	<.0001*	0.91
(>= 14)	100	81.1	5.3	0	52.5	<.0001*	0.91
(>= 15)	100	81.3	5.3	0	52.1	<.0001*	0.91
(>= 16)	100	81.6	5.4	0	51.2	<.0001*	0.91
(>= 17)	100	82.5	5.7	0	48.6	<.0001*	0.91
(>= 18)	100	82.5	5.7	0	48.6	<.0001*	0.91
(>= 19)	100	83.4	6.0	0	46.3	<.0001*	0.92
(>= 20)	100	84.8	6.6	0	42.3	<.0001*	0.92
(>= 21)	100	86.2	7.2	0	38.3	<.0001*	0.93
(>= 22)	95.0	88.2	8.1	0.1	32.3	<.0001*	0.92
(>= 23)	95.0	89.1	8.7	0.1	29.9	<.0001*	0.92
(>= 24)	95.0	89.7	9.2	0.1	28.1	<.0001*	0.92
(>= 25)	72.7	91.8	8.9	0.3	20.0	<.0001*	0.82
(>= 26)	63.4	93.3	9.5	0.4	14.9	0.0001*	0.78
(>= 27)	63.4	93.5	9.8	0.4	14.2	0.0002*	0.78
(>= 28)	63.4	94.2	10.9	0.4	12.4	0.0004*	0.79
(>= 29)	63.4	96.1	16.3	0.4	7.24	0.007*	0.80
(>= 30)	63.4	97.2	22.6	0.4	4.31	0.038*	0.80
(>= 31)	41.0	98.6	29.3	0.6	0.42	0.517	0.70
(>= 32)	31.7	98.9	28.8	0.7	0.03	0.862	0.65

*P-value statistically significant 0.05.
