# Supplementary material for: Accuracy of online survey assessment of mental disorders and suicidal thoughts and behaviors in Spanish university students. Results of the WHO World Mental Health- International College Student initiative
Source: PLoS One. 2019 Sep 5;14(9):e0221529. doi: 10.1371/journal.pone.0221529 (PMC6728025; doi:10.1371/journal.pone.0221529)
Supplement: S8 Table — (PDF) [file pone.0221529.s008.pdf]

S8 Table. Sensitivity, specificity, likelihood ratio positive (LR+), likelihood ratio negative (LR-), McNemar and Area Under the Curve (AUC) for different cut-off points of Generalized Anxiety Disorder lifetime algorithm for estimating reference standard (MINI) (n=287)

Cutpoint	Sensitivity	Specificity	LR+	LR-	McNemar		AUC
					χ^2	p-value	
(>= 13)	100	57.8	2.4	0	115.6	<.0001*	0.79
(>= 14)	100	58.0	2.4	0	114.9	<.0001*	0.79
(>= 15)	100	59.5	2.5	0	110.8	<.0001*	0.80
(>= 16)	100	59.9	2.5	0	109.9	<.0001*	0.80
(>= 17)	100	60.6	2.5	0	107.9	<.0001*	0.80
(>= 18)	100	61.2	2.6	0	106.2	<.0001*	0.81
(>= 19)	100	68.3	3.2	0	86.9	<.0001*	0.84
(>= 20)	100	69.9	3.3	0	82.4	<.0001*	0.85
(>= 21)	100	71.4	3.5	0	78.2	<.0001*	0.86
(>= 22)	97.3	74.8	3.9	0	68.5	<.0001*	0.86
(>= 23)	97.3	77.4	4.3	0	61.3	<.0001*	0.87
(>= 24)	97.3	79.4	4.7	0	55.6	<.0001*	0.88
(>= 25)	85.3	83.4	5.1	0.2	42.2	<.0001*	0.84
(>= 26)	85.3	85.6	5.9	0.2	36.0	<.0001*	0.85
(>= 27)	85.3	86.4	6.3	0.2	34.1	<.0001*	0.86
(>= 28)	64.2	90.4	6.7	0.4	19.5	<.0001*	0.77
(>= 29)	52.6	94.1	8.9	0.5	8.35	0.004*	0.73
(>= 30)	52.6	95.7	12.2	0.5	4.47	0.035*	0.74
(>= 31)	52.6	97.2	18.8	0.5	1.67	0.196	0.75
(>= 32)	35.6	97.8	16.2	0.7	0.19	0.662	0.67

*P-value statistically significant 0.05.
