# Supplementary material for: Accuracy of online survey assessment of mental disorders and suicidal thoughts and behaviors in Spanish university students. Results of the WHO World Mental Health- International College Student initiative
Source: PLoS One. 2019 Sep 5;14(9):e0221529. doi: 10.1371/journal.pone.0221529 (PMC6728025; doi:10.1371/journal.pone.0221529)
Supplement: S9 Table — (PDF) [file pone.0221529.s009.pdf]

S9 Table. Prevalence estimates of common mental disorders and suicidal thoughts and behaviors in the clinical reappraisal samples recruited at each follow-up, according to the WMH-ICS online survey screeners and the MINI (n= 287) (unweighted values)

		Baseline (n= 45)				1[st]Follow-up (n=91)				2[nd]Follow-up (n= 151)			
		Online survey screeners		MINI		Online survey screeners		MINI		Online survey screeners		MINI	
		n	% (SE)	n	% (SE)	n	% (SE)	n	% (SE)	n	% (SE)	n	% (SE)
**Mental disorders**													
**Any mood[a]**	12-m	10	22,2 (0,06)	3	6,7 (0,04)	17	18,7 (0,04)	11	12,9 (0,04)	40	26,5 (0,04)	18	11,9 (0,03)
	Lifetime	11	24,4 (0,06)	9	23,7 (0,06)	40	44 (0,05)	18	21,2 (0,04)	85	57,0 (0,04)	39	26 (0,04)
Major depressive episode	12-m	10	22,2 (0,06)	0	0 (0)	16	17,6 (0,04)	10	11,8 (0,03)	34	22,5 (0,03)	16	10,7 (0,03)
	Lifetime	11	24,4 (0,06)	5	13,2 (0,05)	37	40,7 (0,05)	17	19,8 (0,04)	81	54,4 (0,04)	36	24 (0,03)
Mania/Hypomania	12-m	1	2,2 (0,02)	3	6,7 (0,04)	2	2,2 (0,02)	1	1,1 (0,01)	11	7,3 (0,02)	3	2 (0,01)
	Lifetime	1	2,2 (0,02)	5	12,2 (0,05)	5	5,5 (0,02)	1	1,1 (0,01)	19	12,8 (0,03)	7	4,6 (0,02)
**Any anxiety[b]**	12-m	10	22,2 (0,06)	4	9,5 (0,04)	18	19,8 (0,04)	6	6,6 (0,03)	42	27,8 (0,04)	9	6,1 (0,02)
	Lifetime	10	22,2 (0,06)	4	9,8 (0,04)	31	34,1 (0,05)	10	11 (0,03)	88	58,7 (0,04)	18	12,2 (0,03)
Panic disorder	12-m	2	4,4 (0,03)	3	6,7 (0,04)	1	1,1 (0,01)	5	5,5 (0,02)	2	1,30 (0,01)	9	6 (0,02)
	Lifetime	2	4,4 (0,03)	3	6,7 (0,04)	2	2,2 (0,02)	9	9,9 (0,03)	16	10,9 (0,03)	16	10,6 (0,03)
Generalized anxiety disorder	12-m	9	20 (0,06)	2	4,8 (0,03)	17	18,7 (0,04)	3	3,3 (0,02)	41	27,2 (0,04)	2	1,3 (0,01)
	Lifetime	9	20 (0,06)	3	7,3 (0,04)	30	33 (0,05)	4	4,4 (0,02)	84	56 (0,04)	4	2,7 (0,01)

(*Continued*)

S9 Table

(Continued)

**Suicidal thoughts and behaviors**													
Idea	12-m	5	11,1 (0,05)			4	4,4 (0,02)			19	12,6 (0,03)		
	Lifetime	9	20 (0,06)			25	27,5 (0,05)			64	42,4 (0,04)		
Plan	12-m	6	13,3 (0,05)			7	7,7 (0,03)			14	9,3 (0,02)		
	Lifetime	10	22,2 (0,06)			15	16,5 (0,04)			51	33,8 (0,04)		
Attempt	12-m	1	2,2 (0,02)			0	0 (0)			1	0,7 (0,01)		
	Lifetime	2	4,4 (0,03)			2	2,2 (0,02)			6	4 (0,02)		
Suicidal thoughts and behaviors[c]	12-m	6	13,3 (0,05)	2	4,4 (0,03)	8	8,8 (0,03)	6	6,6 (0,03)	21	13,9 (0,03)	17	12,6 (0,03)
	Lifetime	10	22,2 (0,06)	10	22,2 (0,06)	26	28,6 (0,05)	14	15,4 (0,04)	68	45 (0,04)	47	34,1 (0,04)

a.- Mood include: Major Depression Episode or Mania/Hypomania, assessed with the Composite International Diagnostic Interview Screening Scales [CIDI-SC]; b.- Anxiety include: Panic Disorder or Generalized Anxiety Disorder, assessed with the Composite International Diagnostic Interview Screening Scales [CIDI-SC]; c.- Suicidal thoughts and behaviors based on definition used in Spain suicide prevalence paper (by MJ. Blasco) including: suicidal ideation, suicide plan and suicide attempt (excluding the questions of death wish and non-suicidal self-injury), assessed with question from the Self-Injurious Thoughts and Behaviors Interview [SITBI] and the Columbia-Suicide Severity Rating Scale [C-SSRS]. SE: Standard Error.
