# Supplementary material for: Accuracy of online survey assessment of mental disorders and suicidal thoughts and behaviors in Spanish university students. Results of the WHO World Mental Health- International College Student initiative
Source: PLoS One. 2019 Sep 5;14(9):e0221529. doi: 10.1371/journal.pone.0221529 (PMC6728025; doi:10.1371/journal.pone.0221529)
Supplement: S1 STARD Checklist — (PDF) [file pone.0221529.s010.pdf]

S1 STARD checklist for reporting of studies of diagnostic accuracy (2015 version)

Section & Topic	No	Item	Reported on page #
**TITLE OR ABSTRACT**			
	1	Identification as a study of diagnostic accuracy using at least one measure of accuracy (such as sensitivity, specificity, predictive values, or AUC)	Page 4
**ABSTRACT**			
	2	Structured summary of study design, methods, results, and conclusions (for specific guidance, see STARD for Abstracts)	Page 4
**INTRODUCTION**			
	3	Scientific and clinical background, including the intended use and clinical role of the index test	Page 6-7
	4	Study objectives and hypotheses	Page 7
**METHODS**			
Study design	5	Whether data collection was planned before the index test and reference standard were performed (prospective study) or after (retrospective study)	Page 8
Participants	6	Eligibility criteria	Page 8-9
	7	On what basis potentially eligible participants were identified (such as symptoms, results from previous tests, inclusion in registry)	Page 8-9
	8	Where and when potentially eligible participants were identified (setting, location and dates)	Page 8-9
	9	Whether participants formed a consecutive, random or convenience series	Page 9
Test methods	10a	Index test, in sufficient detail to allow replication	Page 7, 10
	10b	Reference standard, in sufficient detail to allow replication	Page 10-11
	11	Rationale for choosing the reference standard (if alternatives exist)	Page 10-11
	12a	Definition of and rationale for test positivity cut-offs or result categories of the index test, distinguishing pre-specified from exploratory	Page 10, 12
	12b	Definition of and rationale for test positivity cut-offs or result categories of the reference standard, distinguishing pre-specified from exploratory	Page 11,12
	13a	Whether clinical information and reference standard results were available to the performers/readers of the index test	Page 9
	13b	Whether clinical information and index test results were available to the assessors of the reference standard	Page 11
Analysis	14	Methods for estimating or comparing measures of diagnostic accuracy	Page 11-12
	15	How indeterminate index test or reference standard results were handled	Page 12-13
	16	How missing data on the index test and reference standard were handled	Page 12
	17	Any analyses of variability in diagnostic accuracy, distinguishing pre-specified from exploratory	Page 12-13
	18	Intended sample size and how it was determined	Page 12
**RESULTS**			
Participants	19	Flow of participants, using a diagram	Fig 1, page 13
	20	Baseline demographic and clinical characteristics of participants	Table 1, page 14
	21a	Distribution of severity of disease in those with the target condition	---------
	21b	Distribution of alternative diagnoses in those without the target condition	---------
	22	Time interval and any clinical interventions between index test and reference standard	Page 9
Test results	23	Cross tabulation of the index test results (or their distribution) by the results of the reference standard	Table 2, page 16
	24	Estimates of diagnostic accuracy and their precision (such as 95% confidence intervals)	Table 3, page 18
	25	Any adverse events from performing the index test or the reference standard	---------
**DISCUSSION**			
	26	Study limitations, including sources of potential bias, statistical uncertainty, and generalisability	Page 29-30
	27	Implications for practice, including the intended use and clinical role of the index test	Page 31
**OTHER INFORMATION**			
	28	Registration number and name of registry	---------
	29	Where the full study protocol can be accessed	Page 8
	30	Sources of funding and other support; role of funders	Page 3
